# Standard: Human gastric cancer organoids

**DOI:** 10.1186/s13619-024-00217-7

**Published:** 2024-12-27

**Authors:** Ronghui Tan, Fan Hong, Ting Wang, Nanshan Zhong, Hongling Zhao, Rui-hua Xu, Lin Shen, Yingbin Liu, Xuebiao Yao, Dongxi Xiang, Dong Gao, Jianping Xiong, Lijian Hui, Bing Zhao, Zhifeng Miao, Jie Hao, Yong Li, Shijun Hu, Boqiang Fu, Guoqiang Hua, Lei Wang, Zhao-Lei Zeng, Chong Chen, Jianmin Wu, Changlin Wang, Chunnian Wang, Xianbao Zhan, Chen Song, Zhijian Sun, Chunping Yu, Yingying Yang, Gengming Niu, Yalong Wang, Tongbiao Zhao, Ye-Guang Chen

**Affiliations:** 1 Guangzhou National Laboratory, Guangzhou, 510005 China; 2 The State Key Laboratory of Membrane Biology, Tsinghua‑Peking Center for Life Sciences,, School of Life Sciences , Tsinghua University, Beijing, 100084 China; 3 The MOE Basic Research and Innovation Center for the Targeted Therapeutics of Solid Tumors, School of Basic Medical Sciences, Jiangxi Medical College, Nanchang University, Nanchang, 330031 China; 4 Beijing Institute for Stem Cell and Regenerative Medicine, Beijing, 100101 China; 5 State Key Laboratory of Oncology in South China, Collaborative Innovation Center for Cancer Medicine, Sun Yat-Sen University Cancer Center, Guangzhou, 510060 China; 6 Research Unit of Precision Diagnosis and Treatment for Gastrointestinal Cancer, Chinese Academy of Medical Sciences, Guangzhou, 510060 China; 7 State Key Laboratory of Holistic Integrative Management of Gastrointestinal Cancers, Department of Gastrointestinal Oncology, Beijing Key Laboratory of Carcinogenesis and Translational Research, Peking University Cancer Hospital & Institute, Beijing, 100142 China; 8 State Key Laboratory of Systems Medicine for Cancer, Shanghai Cancer Institute, Shanghai Jiaotong University School of Medicine, Shanghai, 200232 China; 9 Department of Biliary-Pancreatic Surgery, Renji HospitalAffiliated to, Shanghai Jiaotong University School of Medicine , Shanghai, 200127 China; 10 MOE Key Laboratory of Cellular Dynamics, CAS Center for Excellence in Molecular Cell Science, University of Science and Technology of China, Hefei, 230027 China; 11 Key Laboratory of Multi-Cell Systems, Shanghai Key Laboratory of Molecular Andrology, Center for Excellence in Molecular Cell Science, Shanghai Institute of Biochemistry and Cell Biology, Chinese Academy of Sciences, Shanghai, 200031 China; 12 University of Chinese Academy of Sciences, Beijing, 100049 China; 13 Institute of Cancer Research, Shenzhen Bay Laboratory, Shenzhen, 518132 China; 14 Department of Oncology, The First Affiliated Hospital of Nanchang University, Nanchang, Jiangxi 330031 China; 15 Jiangxi Key Laboratory for Individual Cancer Therapy, Nanchang, Jiangxi 330031 China; 16 School of Life Science and Technology, Shanghai Tech University, Shanghai, 201210 China; 17 State Key Laboratory of Cell Biology, CAS Center for Excellence in Molecular Cell Science, Shanghai Institute of Biochemistry and Cell Biology, Chinese Academy of Sciences, University of Chinese Academy of Science, Shanghai, 200031 China; 18 Department of Surgical Oncology and General Surgery, The First Hospital of China Medical University, 155N Nanjing Street, Shenyang, Liaoning 110001 China; 19 Key Laboratory of Precision Diagnosis and Treatment of Gastrointestinal Tumors, Ministry of Education, China Medical University, Shenyang, Liaoning 110001 China; 20 Institute of Health Sciences, China Medical University, Shenyang, Liaoning 110001 China; 21 National Stem Cell Resource Center, Institute of Zoology, Chinese Academy of Sciences, Beijing, 100101 China; 22 State Key Laboratory of Stem Cell and Reproductive Biology, Institute for Stem Cell and Regeneration, Institute of Zoology, Chinese Academy of Sciences, Beijing, 100101 China; 23 Department of Gastrointestinal Surgery, Department of General Surgery, Guangdong Provincial People’s Hospital (Guangdong Academy of Medical Sciences), Southern Medical University, Guangzhou, 510080 China; 24 Department of Cardiovascular Surgery of the First Affiliated Hospital & Institute for Cardiovascular Science, Collaborative Innovation Center of Hematology, State Key Laboratory of Radiation Medicine and Protection, Suzhou Medical College, Soochow University, Suzhou, 510080 China; 25 National Institute of Metrology, Beijing, 100029 China; 26 Department of Radiation Oncology and Cancer Institute, Fudan University Shanghai Cancer Center Fudan University, Shanghai, 200433 China; 27 D1Med Technology (Shanghai) Inc, Shanghai, 201802 China; 28 Gastric Cancer Centerand, Laboratory of Gastric Cancer, State Key Laboratory of Biotherapy, West China Hospital, Sichuan University, Chengdu, Sichuan 610041 China; 29 Key Laboratory of Carcinogenesis and Translational Research (Ministry of Education), Center for Cancer Bioinformatics, Peking University Cancer Hospital & Institute, Beijing, 100142 China; 30 Peking University International Cancer Institute, Peking University, Beijing, 100191 China; 31 China National Institute of Standardization, Beijing, 100191 China; 32 Institute of Clinical Science, Zhongshan Hospital, Fudan University, Shanghai, 200433 China; 33 Department of Gastrointestinal Pathology, Ningbo Diagnostic Pathology Center, Ningbo, 315021 China; 34 Department of Oncology, Changhai Hospital, Second Military Medical University, No. 168 Changhai Road, Shanghai, 200433 China; 35 Huayi Regeneration Technology Co., Ltd, Chengdu, 611135 China; 36 K2 Oncology Co., Ltd, KeChuang Street, Beijing, 100176 China; 37 Lilly (China) Research and Development Center, Shanghai, 201203 China; 38 Qilu Pharmaceutical Co., Ltd, Jinan, 250104 China; 39 Shanghai OneTar Biomedicine Co., Ltd, Shanghai, 201203 China

**Keywords:** Standard, Human stomach, Gastric cancer organoids, Terms, Definitions, Technical requirements, Labeling, Transportation, Storage

## Abstract

Gastric cancer is one of the most common malignancies with poor prognosis. The use of organoids to simulate gastric cancer has rapidly developed over the past several years. Patient-derived gastric cancer organoids serve as in vitro models that closely mimics donor characteristics, offering new opportunities for both basic and applied research. The “Human Gastric Cancer Organoid” is part of a series of guidelines for human gastric cancer organoids in China, jointly drafted by experts from the Chinese Society for Cell Biology and its branches, and initially released on October 29, 2024. This standard outlines terminology, technical requirements, assessment protocols, and applies to production, evaluation procedures, and quality control for human gastric cancer organoids. The publication of this guideline aims to assist institutions in endorsing, establishing, and applying best practices, advancing the international standardization of human gastric cancer organoids for clinical development and therapeutic application.

## Scope

This document specifies the ethical requirements, technical requirements, and testing methods, inspection rules, usage instructions, labeling, transportation, and storage for human gastric cancer organoids.

This standard applies to the production and test of human gastric cancer derived organoids.

## Normative references

The following referenced documents are indispensable for the application of these documents. For dated references, only the edition cited applies. For undated references, the latest edition of the referenced document (including all amendments) applies.

*Pharmacopoeia of the People's Republic of China* (2020 Edition, Part III).

## Terms and definitions

The following terms and definitions apply to this document.

### Organoids

Three-dimensional (3D) structures that grow from stem cells or progenitor cells in vitro, are capable of self-organization and renewal, consist of organ-specific cell types and can mimic the in vivo architecture and specific function of the original tissue (Clevers 2016; Kim et al. 2020; Fujii and Sato 2021; Lin et al. 2023).

### Human gastric cancer organoids

Organoids that develop from gastric tumor cells of patients pathologically diagnosed with gastric cancer, and can simulate the characteristics of original gastric cancer tissues (Fujii et al. 2019; Seidlitz et al. 2019; Yan et al. 2018; Wang et al. 2024).

### Organoid passage

Process of dissociating existing organoids into smaller fragments, or single cell via physical, chemical, or biological methods, and keeping them growing in vitro under the same culture conditions (Seidlitz et al. 2019).

### Organoid cryopreservation

Freezing process by which organoids are maintained at low temperature in an inactive state for maintaining cellular composition, gene expression, and functional properties.

### Organoid thawing

Process of bringing frozen organoids from an inactively to an actively growing state.

## General requirements

### Raw materials

The acquisition of raw materials must comply with domestically recognized ethical standards and local laws.

Depending on the intended use, donor evaluation criteria should be established for the research and production of human gastric cancer organoids.

### Process and information management

Critical factors influencing product quality during the procurement, preparation, testing, transportation, and storage should be documented, and a unique identification system should be implemented to ensure traceability throughout the process.

The minimum retention period for records must be clearly defined to ensure the integrity and security of documentation.

## Technical requirements

### Morphology

Human gastric cancer organoids display a variety of morphological characteristics under optical microscopy, including vesicular, compact, and mixed types, among others (Yan et al. 2018; Seidlitz et al. 2019, 2020; Togasaki et al. 2021; Wang et al. 2024;).

### Pathological features

Cells within the organoids should maintain the atypical characteristics of tumor cells found in the original tumor tissue, such as hyperchromatic nuclei, abnormal mitotic figures, and disrupted nuclear-cytoplasmic ratio, etc. (Yan et al. 2018; Seidlitz et al. 2019).

Immunohistochemical testing for relevant markers should be performed on the organoids, and the results should be generally consistent with the original tumor tissues. Markers tested include, but are not limited to, CEA and CA19-9 (Yan et al. 2018; Wang et al. 2024).

### Genetic characteristics

Genetic variation testing should be performed on the organoids, and the results should be essentially the same as the genetic variation results of the original tumor tissue. The tested genes shall include, but not be limited to, *TP53*, *ARID1*, *ARBB2*, *MUC6*, *KRAS*, *RHOA*, *RNF43*, *APC*, *CDH1*, and *PIK3CA*, etc. (Fujii et al. 2019; Togasaki et al. 2021; Schmäche et al. 2024; Wang et al. 2024).

### Culture and growth

Human gastric cancer organoids derived from gastric cancer patient tissues or cells should be capable of being passaged for at least three generations after the initial culture in vitro (Seidlitz et al. 2019, 2020).

Post-passage organoids shall be reconstructed in vitro into new analogous organoids, and their morphology and characteristics shall be consistent with those of the pre-passage organoids (Sato et al. 2011).

### Organoids viability

After thawing, the number of viable gastric cancer organoids should be no less than 50% of the number before cryopreservation, and the viable organoids shall be capable of being subcultured in vitro.

### Microorganisms

Organoids shall be negative for fungi, bacteria, mycoplasma, and virus.

### Identity

The identity of organoids shall match that of the donor tissue by STR analysis (Yang et al. 2024).

## Test methods

### Morphology

Observe organoid morphology by the inverted phase contrast microscope.

### Pathological features

The method can be found in Appendix A.

### Genetic characteristics

The method can be found in Appendix B.

### Culture and growth

Organoids cultured in vitro can be photographed using optical microscopy, with a scale bar for measuring their diameter and performing quantitative analysis.

### Organoids viability

Organoids viability shall be counted according to the method can be found in Appendix C.

### Microorganisms

#### Bacteria and Fungi

The “1101 Sterility Inspection Method” in *Pharmacopoeia of the People's Republic of China* (2020 Edition, Part III) shall be followed.

#### Mycoplasma

The “3301 Mycoplasma Inspection Method” in *Pharmacopoeia of the People's Republic of China* (2020 Edition, Part III) shall be followed.

#### Exogenous viral factors

The “3302 Exogenous Viral Factors Inspection Method” in *Pharmacopoeia of the People's Republic of China* (2020 Edition, Part III) shall be followed.

### STR

The method can be found in Appendix D.

## Instructions for use

The instructions should include at least the following information:

- Organoid code;
- Passage number;
- Organoid number;
- Production date;
- Batch number;
- Manufacturing organization;
- Storage conditions;
- Transportation conditions;
- Contact information;
- Usage instructions;
- Standard reference number;
- Production address;
- Postal code;
- Precautions.

Note: Endotoxin results should be provided upon user request.

## Labeling

The labels should include at least the following information:

- Organoid code;
- Passage number;
- Organoid number;
- Batch number;
- Manufacturing organization;
- Production date.

## Transportation and storage

### Transportation

The transportation methods and conditions should be selected based on the requirements for the use of human gastric cancer organoids to ensure their biological properties, safety, stability, and efficacy.

The transportation of human gastric cancer organoids should consider, but not be limited to, factors such as the characteristics of the organoids, the container carrying the organoids, transportation route, conditions, equipment, methods, potential risks, and necessary safeguards.

The control measures for transportation conditions should include, but not be limited to, temperature range, vibration control, contamination prevention, equipment performance, and appropriate packaging.

Relevant inspection and technical guidance documents should be provided upon the user’s request.

The package should be checked during transportation, and if necessary, additional freezing sources (e.g., dry ice and liquid nitrogen) should be added to maintain the appropriate transportation temperature.

### Storage

Optimized cryopreservation protocols and methods should be employed to minimize damage to human gastric cancer organoids during freezing and thawing processes, ensuring their normal functionality is minimally affected.

The cryopreservation information for human gastric cancer organoids should be documented, including but not limited to:

- Organoid code;
- Batch number;
- Organoid number;
- Passage number;
- Freezing date;
- Cryoprotectant composition;
- Name of the operator.

The storage conditions for human gastric cancer organoids should be documented, including but not limited to:

- Storage conditions;
- Storage date;
- Storage duration;
- Storage personnel.

## Data Availability

All data needed to evaluate the conclusions in the paper are present in the paper.

## Appendix A

### Organoid Histopathology Test (Paraffin Embedding Method)

#### A.1 Instruments

*A.1.1* Paraffin-embedding machine

*A.1.2* Paraffin-slicer

#### A.2 Reagents

Unless otherwise specified, the reagents used shall be analytically pure, and the water used for testing shall be deionized water.

*A.2.1* Paraffin section preparation reagents: prepare reagents required for paraffin embedding according to the corresponding requirements, including fixing solution, dehydration solution, paraffin, dewaxing solution, rehydration solution, ethanol, xylene, and neutral resin.

*A.2.2* H&E staining reagent: hematoxylin, eosin.

*A.2.3* Immunohistochemical staining reagents: prepare antigen repair solution, primary antibody, secondary antibody, blocking buffer, Phosphate buffer saline (PBS), DAB according to the corresponding requirements.

#### A.3 Testing protocol

##### A.3.1 Sample preparation and fixation

The organoids are mechanically pipetted out of Matrigel gently and transferred to a 15 mL centrifuge tube. Collect the organoids by centrifugation and discard the supernatant. The organoids are fixed with 4% paraformaldehyde for 15~30 minutes.

##### A.3.2 Paraffin section preparation of organoids

The organoid samples are fixed, dehydrated, hyalinized, immersed in paraffin, and with a paraffin-embedding machine according to the paraffin-section method. Cut the embedded-paraffin of organoids into standard thickness with paraffin-slicer.

##### A.3.3 H&E staining

Paraffin sections of organoids are dewaxed, rehydrated, stained with hematoxylin and eosin, then dehydrated with ethanol, hyalinized by xylene, and sealed by neutral resin.

##### A.3.4 Immunohistochemistry staining

Paraffin sections of organoids are dewaxed, rehydrated, antigen repaired and sealed with blocking solution, and sections are incubated with primary antibody and then cleaned with PBS, followed by incubated with second antibody and cleaned with PBS. Perform the color reaction with DAB and add water to stop the color reaction. The sections are dyed by hematoxylin, hyalinized with hydrochloric acid and flushed, and then dehydrated with ethanol, hyalinized by xylene, and sealed by neutral resin.

#### A.4 Result analysis

The test results obtained are analyzed and judged by personnel qualified for pathological diagnosis, and these results shall be consistent with the results of the original tumor tissue.

## Appendix B

### Organoid Gene Mutation Test

#### B.1 Instruments

*B.1.1* Centrifuge

#### B.2 Reagents

Cell DNA extraction kit.

#### B.3 Sample storage

The samples are prepared and stored below -80 ℃.

#### B.4 Testing Protocol

##### B.4.1 Sample preparation

The organoids are cultured in the Matrigel to a stable growth state, and then mechanically pipetted out of the Matrigel. The mixture is collected in a centrifugal tube, the organoids are collected by centrifugation, and the supernatant is discarded.

##### B.4.2 Extraction of DNA

Perform genomic DNA extraction from organoids and primary tumor tissues according to the instructions of the Cell DNA extraction kit.

##### B.4.3 DNA sequencing

Send the organoid DNA samples and original tumor tissues to institutions qualified for clinical genetic testing for 1^st^ or 2^nd^ generation sequencing testing, or the genetic loci will be tested by amplification refractory mutation system (ARMS) method or droplet digital PCR method.

#### B.5 Result analysis

Analyze the mutant loci and compare the concordance between organoid and original tumor tissue sequencing results.

## Appendix C

### Organoids Viability (Calcein-AM Staining Method)

#### C.1 Instruments

*C.1.1* Inverted microscope.

*C.1.2* Fluorescence microscope.

#### C.2 Reagents

Unless otherwise specified, the reagents used shall be analytically pure, and the water used for testing shall be deionized water.

*C.2.1* Dimethyl sulfoxide (DMSO) for cell culture.

*C.2.2* PBS (pH 7.4).

*C.2.3* Storage of Calcein-AM solution: 2 mmol/L in DMSO.

#### C.3 Testing protocol

##### C.3.1 Organoid observation in bright field

Place the organoids under the microscope to observe their morphology and status. Determine whether the organoid morphology meets the requirements of 6.1 by visual observation.

##### C.3.2 Organoid quantification

Add the Calcein-AM storage solution to the medium until the final concentration is 0.2 μmol/L, and incubate the mixture for 60 minutes at 37 ℃. Then clean the medium with Calcein-AM slowly with PBS and add fresh medium. The organoids are observed and photographed by fluorescence microscope at 490 nm excitation wavelength and 515 nm emission wavelength. Living organoids are in green with clear edges. Count the number of organoids with a diameter ≥20 μm.

#### C.4 Accuracy

The absolute difference between the results of three independent determinations obtained under reproducible conditions shall not exceed 10% of the arithmetic mean.

## Appendix D

### Organoid Authentication by STR Profile

#### D.1 Instruments

*D.1.1* Centrifuge.

*D.1.2* PCR-Cycler.

*D.1.3* Electrophoresis apparatus.

*D.1.4* Microvolume UV Spectrophotometer.

#### D.2 Reagents

*D.2.1* Cell DNA extraction kit.

*D.2.2* STR DNA profiling kit.

#### D.3 Sample storage

The samples are prepared and stored below -80 ℃.

#### D.4 Testing protocol

##### D.4.1 Sample preparation

The organoids are cultured in the Matrigel to a stable growth state, and then mechanically pipetted out of the Matrigel. The mixture is collected in a centrifugal tube, the organoids are collected by centrifugation, and the supernatant is discarded.

##### D.4.2 Extraction of DNA

A) Perform genomic DNA extraction from organoids and primary tumor tissues according to the instructions of the Cell DNA extraction kit.
B) Measure the absorbance of extracted DNA by UV spectrophotometer to ensure that the ratio of A260/A280 is between 1.8 and 2.0.
C) DNA volume ≥ 20 μL, DNA concentration ≥ 50 ng/μL.

##### D.4.3 PCR amplification

A) Perform STR DNA amplification according to standard PCR amplification methods or the commercially approved kit instructions.
B) Use sterile water as the template for PCR amplification in the negative control group; use the DNA extracted from organoid and primary tumor tissue samples as a template for PCR amplification in the sample detection group; use a DNA template with a confirmed STR profiling as the positive control group.
C) Detect the PCR products of three groups by agarose gel electrophoresis. Clear target band shall be observed in the positive control but not in the negative control.

##### B.4.4 STR genotyping

Detect PCR products by capillary electrophoresis gene analyzer and STR genetic map data are obtained. The PCR banding pattern of organoids and primary tumor tissue shall be consistent.

#### D.5 Result analysis

*D.5.1* When STR alleles contain the same number of repeats, only one allele peak shall appear in the profile, when they contain different numbers of repeats, two allele peaks appear in the profile. The test is considered valid when no allele peaks appeared in the negative control group and the positive control group is consistent with its standard genotyping data.

*D.5.2* If more than two allelic peaks are present at the STR locus of the tested sample, the sample shall be determined to be cross-contaminated after repeated experiments to exclude interfering factors such as mutations in the primer binding region, provided that the test is valid.

## Abbreviations

3D: Three Dimension
DAB: Diaminobenzidine
DMSO: Dimethyl Sulfoxide
DNA: Deoxyribonucleic Acid
H&E: Hematoxylin and Eosin
PBS: Phosphate Buffer Saline
PCR: Polymerase Chain Reaction
STR: Short Tandem Repeat
